# Supplementary figures and images for: Differential sustained and transient temporal processing across visual streams
Source: PLoS Comput Biol. 2019 May 30;15(5):e1007011. doi: 10.1371/journal.pcbi.1007011 (PMC6583966; doi:10.1371/journal.pcbi.1007011)

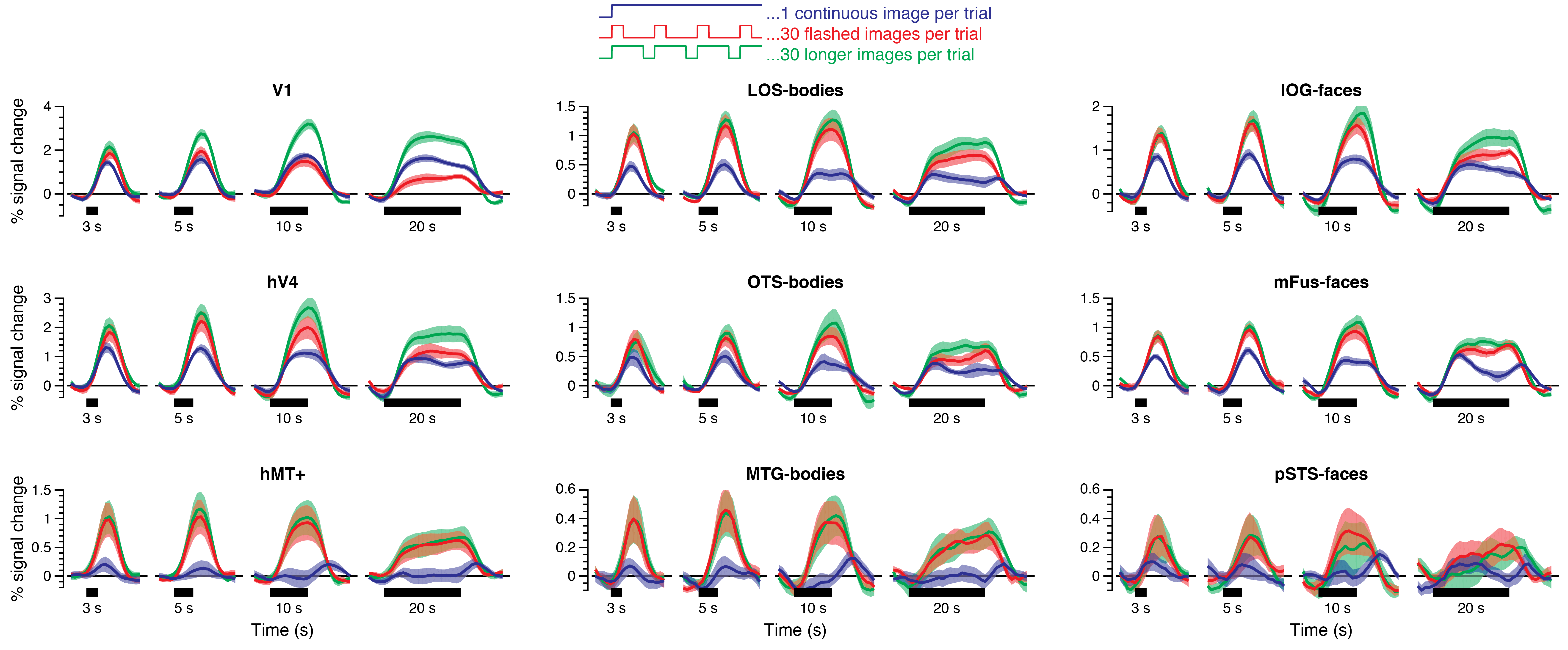

Supplement: S1 Fig — Measured responses in occipital (V1, LOS-bodies, IOG-faces), ventral (hV4, OTS-bodies, mFus-faces), and lateral (hMT+, MTG-bodies, pSTS-faces) regions of interest in experiment 1 (blue), experiment 2 (red), and experiment 3 (green) averaged across all three stimulus categories. Lines: mean response time series across participants; shaded areas: standard error of the mean (SEM) across participants; Horizontal black bars: trial duration. (TIF) [file pcbi.1007011.s001.tif]

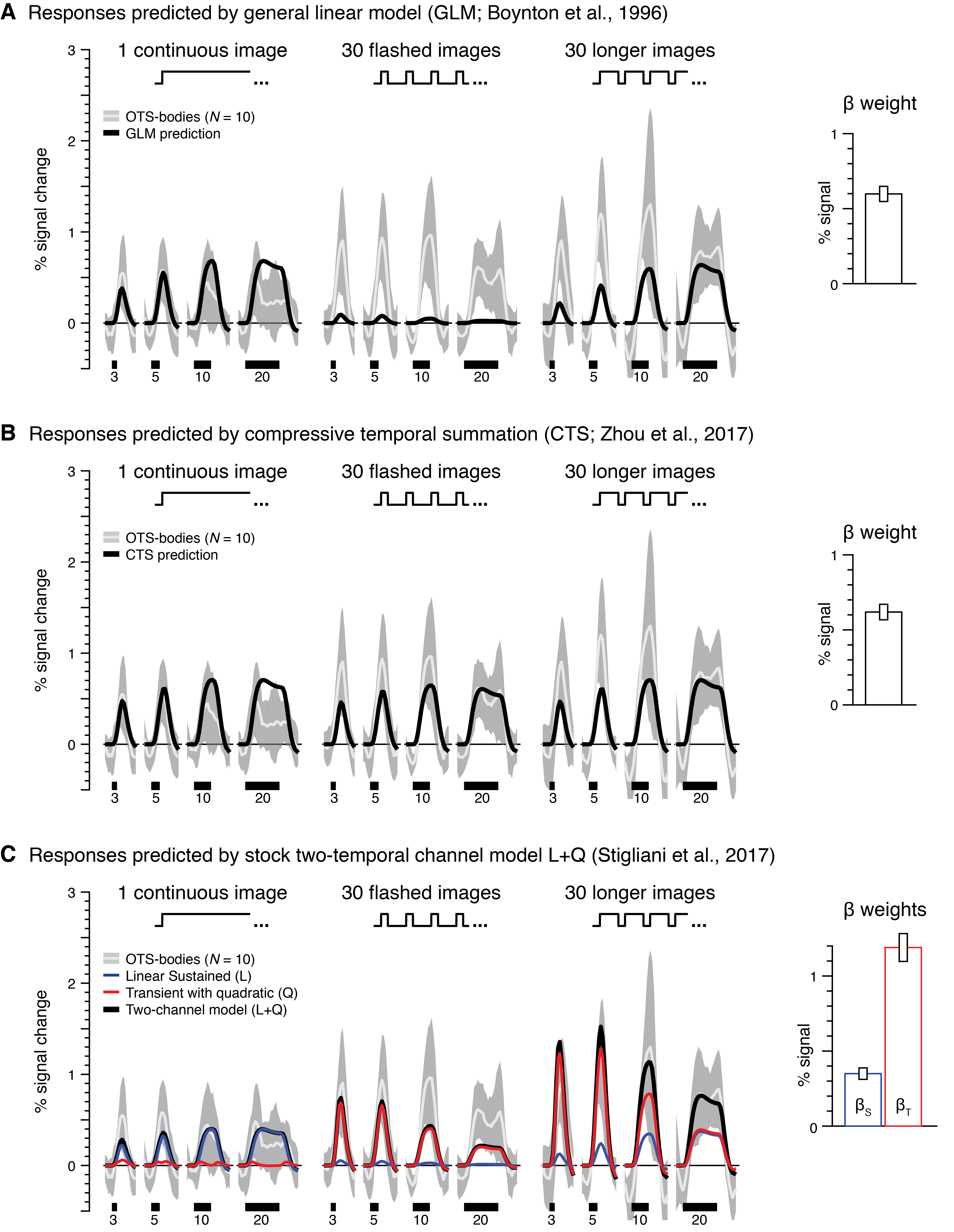

Supplement: S2 Fig — (A-C) Responses and model predictions for body images in OTS-bodies for each experiment (left) with estimated β weights for each model (right). White curve: mean response across 10 participants. Shaded gray: standard deviation across participants. Black curve: overall model prediction. Horizontal black bar: trial duration. (A) Predictions of a general linear model (GLM) [8]. (B) Predictions of a model with compressive temporal summation (CTS) [6]. (C) Predictions of the two-temporal channel L+Q model with linear sustained channel and quadratic transient channel. Blue curve: predicted response from the sustained channel. Red curve: predicted response from the transient channel: Black curve: sum of responses from both channels. In the continuous (left) and flashed images (middle) experiments the model’s prediction (black) is obscured by the response of a single channel, as the other channel’s contribution is negligible. (TIF) [file pcbi.1007011.s002.tif]

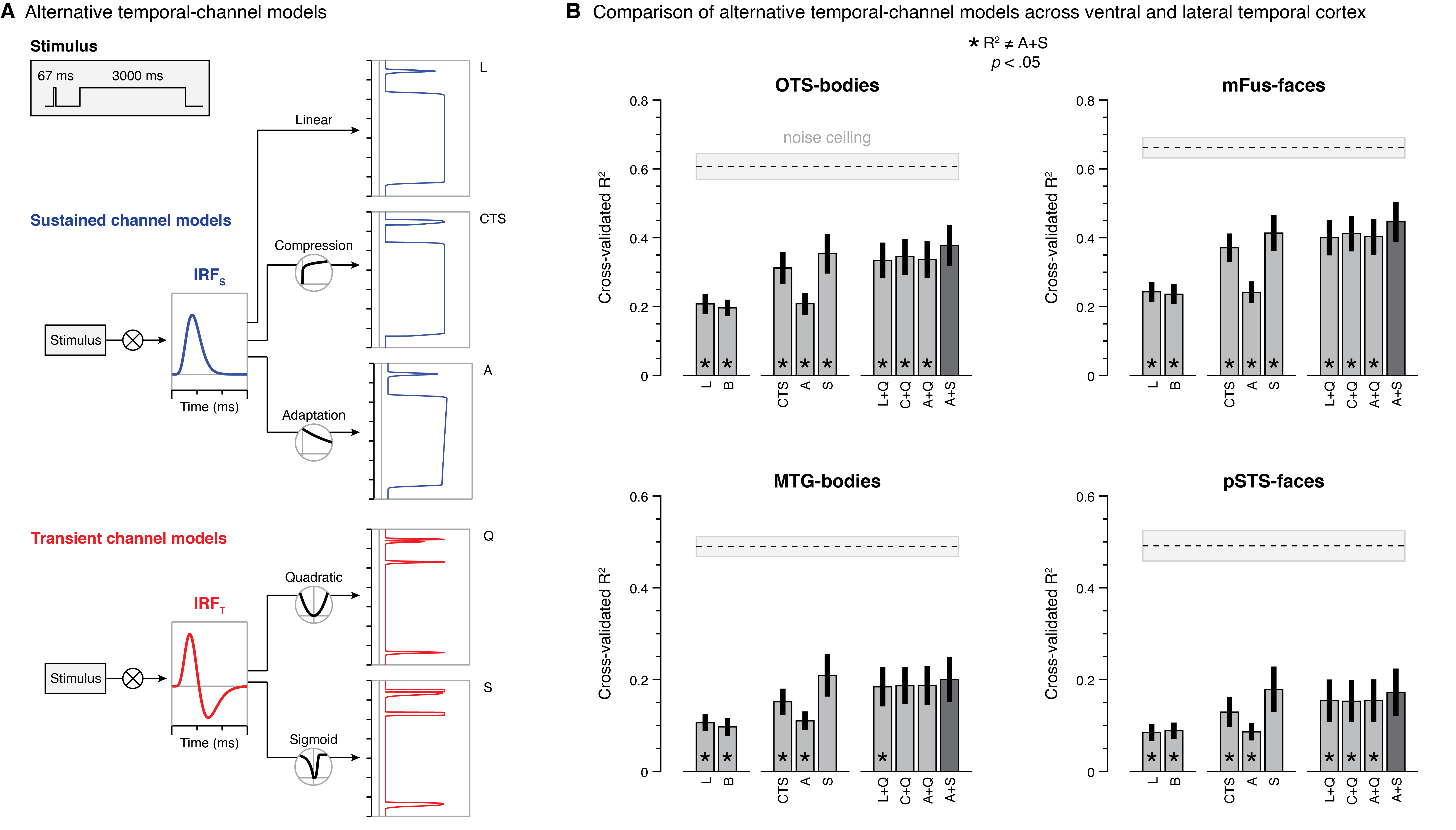

Supplement: S3 Fig — (A) Alternative models of sustained (blue) and transient (red) channels. Schematic depicts neural response predictions generated by different implementations of each channel for both a brief (67 ms) and long (3 s) stimulus. Sustained channel models: L, a linear sustained channel; CTS, a sustained channel with compressive temporal summation [6]; A, a sustained channel with adaptation [8]. Transient channel models: Q, a transient channel with a quadratic (squaring) nonlinearity; S, a transient channel with a sigmoid nonlinearity. (B) Comparison of model performance (cross-validated R2) in each region averaged across all three experiments. Hemodynamic models: L, same as in (a); B, balloon model [7]. Single-channel neural models: CTS, A, and S, same as in (a). Two-channel neural models: L+Q, a linear sustained channel and a transient channel with a quadratic nonlinearity [5]; C+Q, a sustained channel with compressive temporal summation and a transient channel with a quadratic nonlinearity; A+Q, a sustained channel with adaptation and a transient channel with a quadratic nonlinearity; A+S, a sustained channel with adaptation and a transient channel with a sigmoid nonlinearity. Cross-validated R2 significantly differs across models in all four regions (significant main effect of model type, Fs > 6.80, Ps < .001, one-way repeated measures ANOVA for each region). Asterisks denote models with significantly different performance compared to the A+S model, p<0.05. (TIF) [file pcbi.1007011.s003.tif]

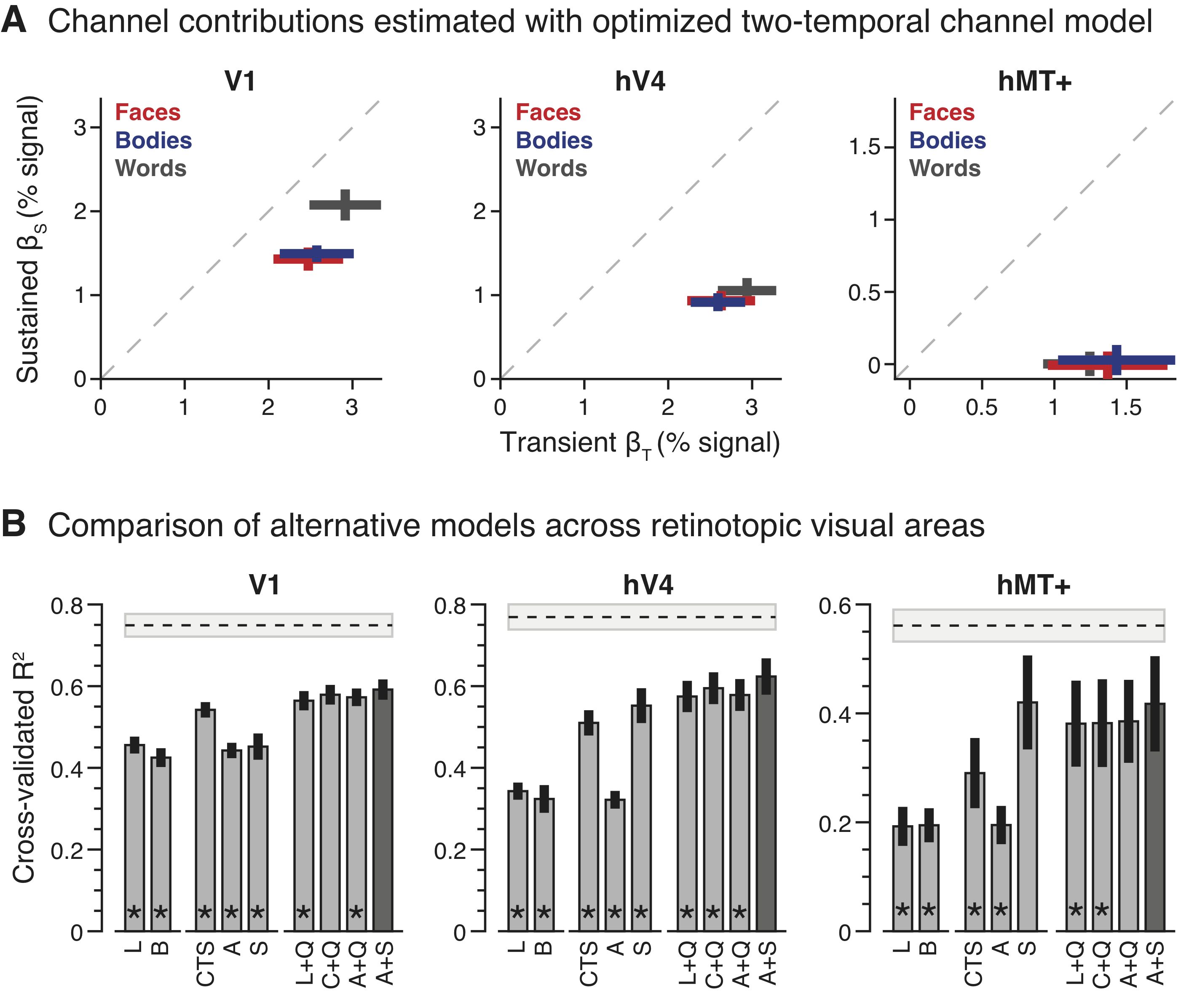

Supplement: S4 Fig — (A) Contributions (β weights) of transient (x axis) and sustained (y axis) channels for each stimulus category estimated by the two-temporal channel A+S model in V1, hV4, and hMT+. Crosses span ±1 SEM across participants in each axis, and β were solved by fitting the model using data concatenated across all experiments. Data show average model weights across all splits of the data for each participant. Red: response to faces. Blue: response to bodies. Gray: response to words. Dashed gray: identity line (βS = βT). (B) Comparison of model performance (cross-validated R2) in each region averaged across all three experiments. Hemodynamic models: L and B. Single-channel neural models: CTS, A, and S. Two-channel neural models: L+Q [5], C+Q, A+Q, and A+S. Cross-validated R2 significantly differs across models in all three regions (significant main effect of model type, Fs > 16.45, Ps < .001, one-way repeated measures ANOVA for each region). Asterisks denote models with significantly different performance vs. the A+S model. (TIF) [file pcbi.1007011.s004.tif]

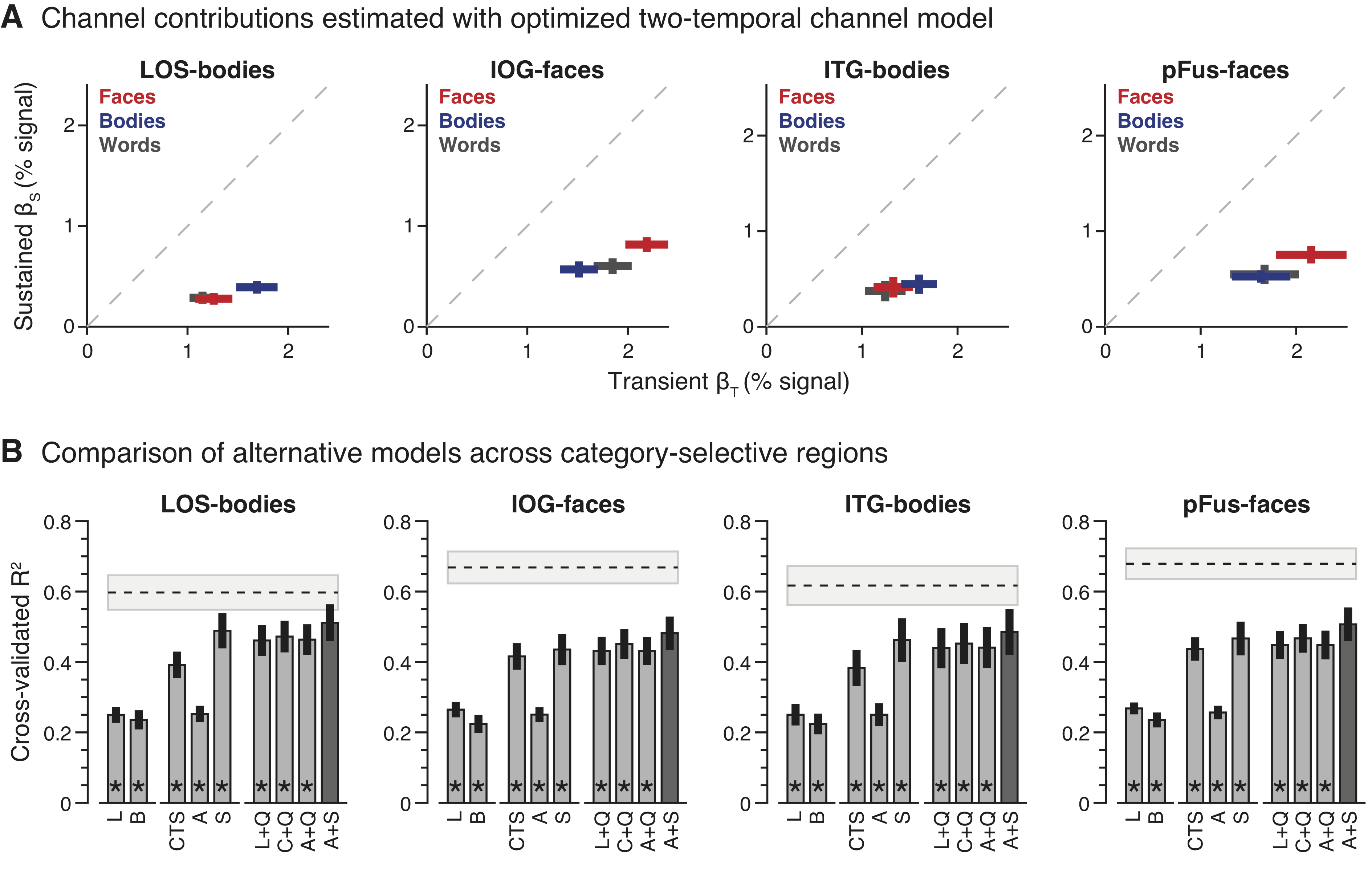

Supplement: S5 Fig — (A) Contributions (β weights) of transient (x axis) and sustained (y axis) channels for each stimulus category estimated by the two-temporal channel A+S model. Crosses span ±1 SEM across participants in each axis, and β were solved by fitting the model using data concatenated across all experiments. Data show average model β weights across all splits of the data for each participant. Red: response to faces. Blue: response to bodies. Gray: response to words. Dashed gray: identity line (βS = βT). (B) Comparison of model performance (cross-validated R2) in each region averaged across all three experiments. Hemodynamic models: L and B. Single-channel neural models: CTS, A, and S. Two-channel neural models: L+Q [5], C+Q, A+Q, and A+S. Cross-validated R2 significantly differs across models in all four regions (significant main effect of model type, Fs > 36.00, Ps < .001, one-way repeated measures ANOVA for each region). Asterisks denote models with significantly different performance vs. the A+S model, p<0.05. (TIF) [file pcbi.1007011.s005.tif]

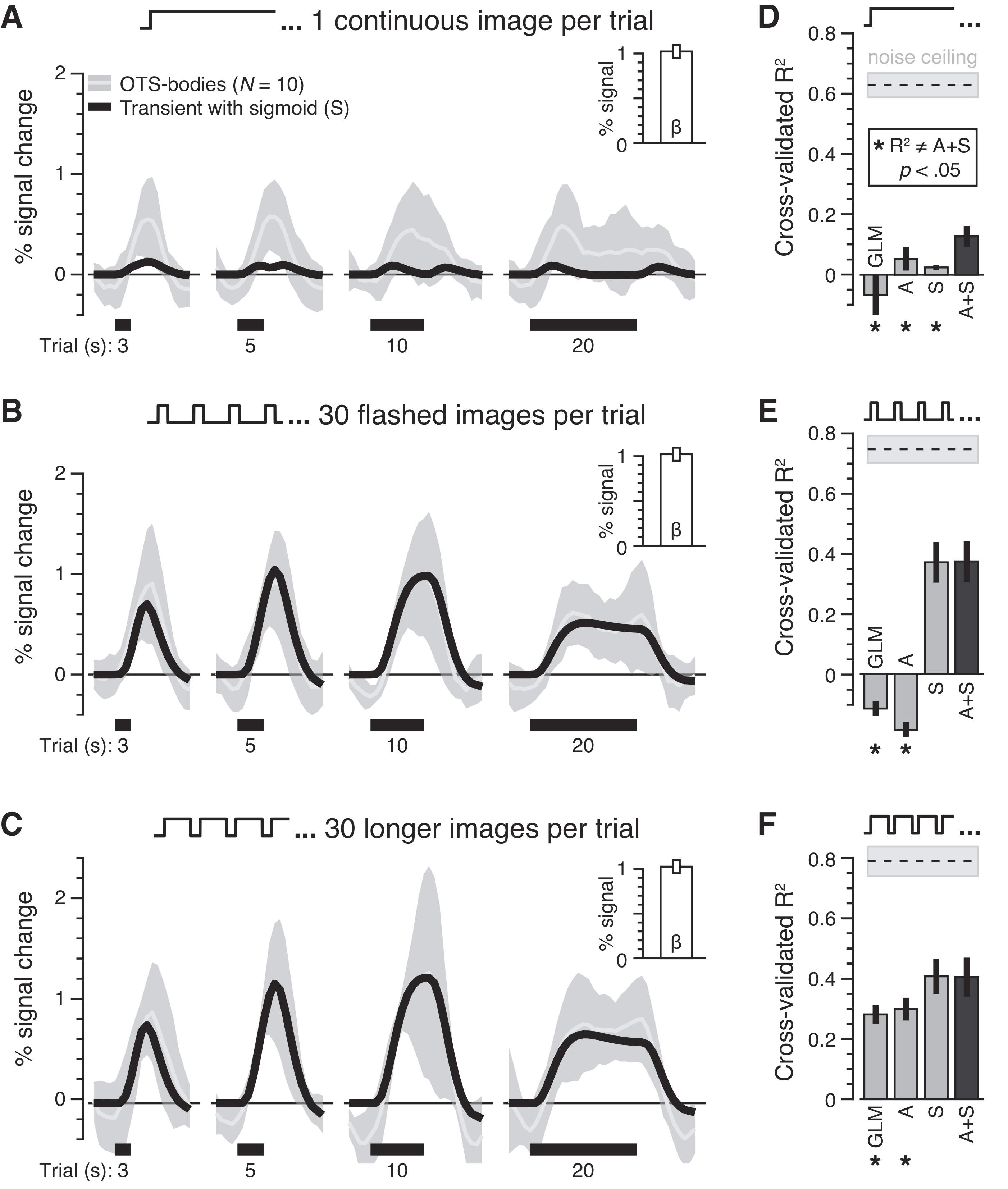

Supplement: S6 Fig — (A-C) Responses for body images in OTS-bodies and predictions of a model with a transient channel, but no sustained channel. White curve: mean response across 10 participants. Shaded gray: standard deviation across participants. Black: predicted response from the transient channel: Inset: mean contribution (β weight) for the transient channel ±1 SEM across participants. (A) Experiment 1 data, 1 continuous image per trial. (B) Experiment 2 data, 30 flashed images per trial. (C) Experiment 3 data, 30 longer images per trial. (D-F) Model comparison. Bars show the performance of various models for each experiment presented in (A-C). Models are fit using runs from all three experiments, and cross-validation performance (x-R2) is calculated in left-out data from each experiment separately. (D) Experiment 1. (E) Experiment 2. (F) Experiment 3. Single-channel models: GLM, general linear model [8]; A, a sustained channel with adaptation; S, a transient channel with a sigmoid nonlinearities; A+S: a sustained channel with adaptation and a transient channel with sigmoid nonlinearities. Cross-validated R2 significantly differs across models [significant main effect of model type, F6, 54 = 21.88, P < .001, two-way repeated measures ANOVA with factors of model type (GLM/A/S/A+S) and experiment (1/2/3)]. Asterisks denote models with significantly different performance compared to A+S (paired t-tests comparing x-R2 of each model vs. A+S in each experiment). (TIF) [file pcbi.1007011.s006.tif]

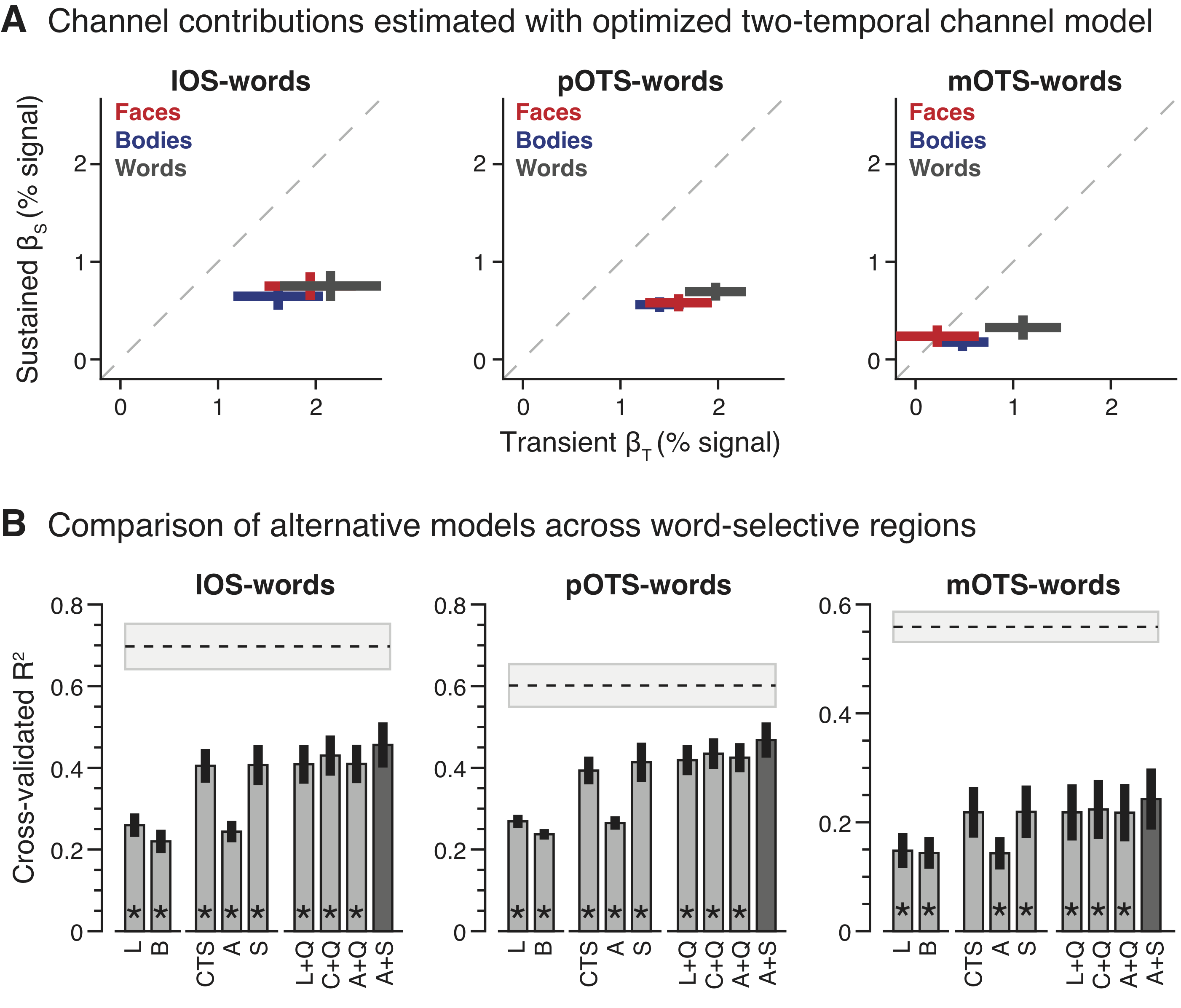

Supplement: S7 Fig — (A) Contributions (β weights) of transient (x axis) and sustained (y axis) channels for each stimulus category estimated by the two-temporal channel A+S model in additional word-selective regions. Crosses span ±1 SEM across participants in each axis, and β were solved by fitting the model using data concatenated across all experiments. Data show average model weights across all splits of the data for each participant. Red: response to faces. Blue: response to bodies. Gray: response to words. Dashed gray: identity line (βS = βT). (B) Comparison of model performance (cross-validated R2) in each region averaged across all three experiments. Hemodynamic models: L and B. Single-channel neural models: CTS, A, and S. Two-channel neural models: L+Q [5], C+Q, A+Q, and A+S. Cross-validated R2 significantly differs across models in all three regions (significant main effect of model type, Fs > 10.17, Ps < .001, one-way repeated measures ANOVA for each region). Asterisks denote models with significantly different performance vs. the A+S model, <0.05. (TIF) [file pcbi.1007011.s007.tif]
